# 伊曲茶碱中间体杂质的分离与鉴定

**DOI:** 10.3724/SP.J.1123.2020.10013

**Published:** 2021-04-08

**Authors:** Yiyun WANG, Xiaofang LÜ, Haojie XU, Zihu MENG, Jiarong LI, Zhibin XU, Min XUE

**Affiliations:** 1.北京理工大学化学与化工学院, 北京 102488; 1. School of Chemistry and Chemical Engineering, Beijing Institute of Technology, Beijing 102488, China; 2.山东新华制药股份有限公司, 山东 淄博 255086; 2. Shandong Xinhua Pharmaceutical Co., Ltd., Zibo 255086, China

**Keywords:** 高效液相色谱, 核磁共振, X射线衍射, 伊曲茶碱, 杂质, 帕金森氏病, high performance liquid chromatography (HPLC), nuclear magnetic resonance (NMR), X-ray diffraction (XRD), istradefylline, impurity, Parkinson’s disease

## Abstract

伊曲茶碱是一种新型选择性腺苷A_2A_受体拮抗剂,用于治疗帕金森氏病和改善帕金森氏病初期运动障碍。在伊曲茶碱中间体A1(6-氨基-1,3-二乙基-2,4-(1*H*,3*H*)-嘧啶二酮)的合成过程中,碱性条件或高温条件下会伴随生成至少2种副产物,在前期研究中我们已经对该中间体合成过程中的其中一种副产物进行过研究,确定其结构为(*E*)*-N*-乙基-2-氰基-3-乙氨基-2-丁烯酰胺。本文采用高效液相色谱(HPLC)对中间体A1的另外一种杂质进行分析:称取0.4 g中间体放入50 mL的烧杯中,依次加入8 mL水、8 mL乙腈,超声溶解,经0.45 μm有机膜过滤,得到测试样品溶液。采用Agilent Zorbax C18色谱柱(150 mm×4.6 mm, 5 μm)分离,柱温35 ℃,流动相为乙腈(A)和水(B),梯度洗脱(*t*_min_/A∶B)=*t*_0_/20∶80, *t*_15_/60∶40, *t*_20_~*t*_50_/90∶10;流速1.0 mL/min;检测波长268 nm。然后通过Ceres B制备色谱柱分离,以乙腈-水(30/70, v/v)为流动相,流速为30 mL/min,在268 nm波长下检测,洗脱得到杂质纯品。通过高分辨率质谱(HRMS)、一维核磁共振(NMR)、二维核磁共振(2D NMR)对杂质进行了结构确认,并通过单晶X射线衍射(XRD)进行了表征。杂质经分析确认为1-(1,3-二乙基-2,6-二氧-1,2,3,6-四氢嘧啶-4-基)-3-乙基脲。根据杂质的化学结构推测其生成机理为:在碱性条件或高温条件下合成中间体A1时,过量的二乙基脲继续与中间体A1发生酰胺化反应而得到此副产物。此杂质与伊曲茶碱中间体A1结构相似,会伴随A1参与到伊曲茶碱合成的后续反应中,并对伊曲茶碱的安全性和有效性产生潜在的影响。因此,为了确保伊曲茶碱的质量,在生产过程中需要对该杂质的含量进行控制。

## Abstract (en)

Istradefylline is a novel selective adenosine A_2A_ receptor antagonist that is used to treat Parkinson’s disease and improve motor dysfunction in the early stage of this disease. During the synthesis of intermediate A1 (6-amino-1,3-diethyl-2,4-(1*H*,3*H*)-pyrimidinedione), at least two by-products were formed under alkaline or high-temperature conditions. In a previous study, one of the by-products in the synthesis of the intermediate was studied, and its structure was identified as (*E*)*-N*-ethyl-2-cyano-3-ethylamino-2-butene amide. In this study, we used high performance liquid chromatography (HPLC) to analyze another impurity formed during the synthesis of A1, and the following steps were executed: 0.4 g of intermediate was weighed and added to a 50 mL beaker, followed by the sequential addition of 8 mL water and 8 mL acetonitrile, and then, ultrasonic dissolution was performed. Finally, the solution was filtered through a 0.45-μm organic membrane and the test sample solution was obtained. We used the Agilent zorbax C18 chromatography column, with acetonitrile (A)/water(B) as the mobile phase under gradient elution ((*t*_min_/A∶B)=*t*_0_/20∶80, *t*_15_/60∶40, *t*_20_-*t*_50_/90∶10). The detector wavelength is 268 nm. In order to separate the impurity from A1, we used a Ceres B preparative column, with acetonitrile-water (30/70, v/v) as the mobile phase. The flow rate was set at 30 mL/min, and the detection wavelength was 268 nm. The structure of the impurity was confirmed by high-resolution mass spectrometry (HRMS), one-dimensional nuclear magnetic resonance (NMR), and two-dimensional nuclear magnetic resonance (2D NMR), and characterized by single-crystal X-ray diffraction (XRD). In MS experiments, an electrospray ionization (ESI) source was used with positive ion scanning. In the NMR experiments, we used tetramethylsilane (TMS) as the internal standard and deuterated dimethyl sulfoxide (DMSO-d_6_) as the solvent to obtain the spectra. The results of preparative high performance liquid chromatography (Prep-HPLC) showed that good separation effect could be achieved by isocratic elution, and the impurity was perfectly separated. The^1^H-NMR spectral data are as follows:^1^H-NMR (600 MHz, DMSO): *δ* 1.01 (q, *J*=6.9 Hz, 3H), 1.02 (q, *J*=6.9 Hz, 3H), 1.07 (t, *J*=6.9 Hz, 3H), 3.04 (p, *J*=6.8 Hz, 2H), 3.74 (q, *J*=7.0 Hz, 2H), 3.94 (q, *J*=7.1 Hz, 2H), 5.85 (s, 1H). The ^13^C-NMR spectral data are summarized as follows: ^13^C-NMR (150 MHz, DMSO): *δ*13.9, 14.1, 15.9, 34.6, 34.9, 36.9, 81.9, 152.2, 153.3, 159.3, 162.0. The impurity was characterized by single-crystal XRD, and its spatial structure was further verified and determined as 1-(1,3-diethyl-2,6-dioxo-1,2,3,6-tetrahydropyrimidin-4-yl)-3-ethylurea. Based on the chemical structure of the impurity, we propose the following mechanism for the impurity: when A1 is synthesized under alkaline conditions or at high temperature, excessive diethylurea continues to undergo amidation with A1 to obtain this by-product. Although the formation mechanism of the impurity studied in this paper is different from that of the intermediate A1 impurity (*E*)*-N*-ethyl-2-cyano-3-ethylamino-2-butene amide, both the impurities are formed at high temperatures. Both will be accompanied by A1 in the subsequent reaction of istradefylline synthesis. The relationship between drug impurities and drug safety is a complex relationship that is affected by many factors. Generally, most impurities in drugs have potential biological activities, and some even interact with the drugs, thus affecting their efficacy and safety and inducing toxic effects. Therefore, to ensure the quality of istradefylline, it is necessary to control the impurity content during the production. The findings of this paper may provide guidelines for controlling the impurity content in istradefylline.

帕金森氏病(PD)是老年人的神经退行性疾病。该疾病主要导致运动障碍,如静息性震颤、肌肉僵硬、运动迟缓和步态异常,以及非运动性症状,如肌张力低下、睡眠障碍、焦虑和抑郁^[1,2]^。PD影响世界各地的数百万人。中国有超过250万PD患者。研究表明,PD的发病率会随着年龄的增长而显著增加,到2030年,PD的患者数量可能会增加一倍^[3,4]^。目前,尚未发现可以逆转或治愈PD的确切药物和相应疗法。因此,如何有效地延迟PD的进程是当前亟待解决的问题。PD的治疗主要是临床对症治疗为主,以康复治疗为辅,其中药物治疗是主要治疗手段^[5,6]^。左旋多巴(L-dopa)是控制PD的非常有效的代表性药物,但长期大量服用左旋多巴常伴有药物运动型并发症剂末现象,导致PD症状加重或者恶化^[7,8]^。

由日本的Kyowa Hakko Kogyo公司研发的伊曲茶碱((*E*)-8-(3,4-二甲氧基苯乙烯基)-1,3-二乙基-7-甲基-3,7-二氢-1*H*-嘌呤-2,6-二酮,见图1)是一种新型选择性腺苷A_2A_受体拮抗剂,通过激活丘脑底核的*γ*-氨基丁酸(GABA)能通路,改善患者的运动症状^[9]^。

**图 1:**
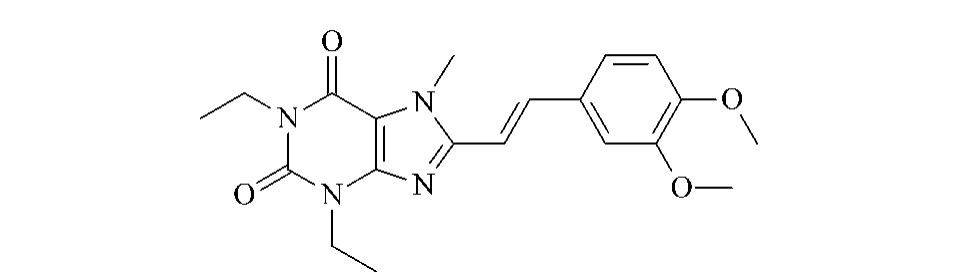
伊曲茶碱的化学结构

伊曲茶碱于2013年3月在日本上市,作为左旋多巴/卡比多巴的辅助疗法,能明显缩短关期,延长开期,可显著降低左旋多巴治疗的剂末现象^[10,11,12,13]^。与其他抗PD药物相比,伊曲茶碱具有较小的副作用,在克服多巴胺药物的某些缺陷上具有显著作用,因此在治疗PD方面具有广阔的前景^[14]^。

伊曲茶碱的上市在PD患者的药物治疗上又迈出了成功的一步,但在伊曲茶碱的合成过程中,发现其中还存在一些未知杂质,这些杂质的存在会对药物活性带来一定的影响,对安全性带来一定的风险。因此鉴定和减少药物中的杂质非常必要,可以保证药物的有效性和安全性^[15,16]^。目前高效液相色谱(HPLC)是最常用的杂质检测方法之一^[17,18]^。

在伊曲茶碱合成中,中间体A1(6-氨基-1,3-二乙基-2,4-(1*H*,3*H*)-嘧啶二酮)是其中一个关键中间体,由1,3-二乙基脲与氰基乙酸经缩合、环合反应制得^[19]^(见图2)。

**图 2:**
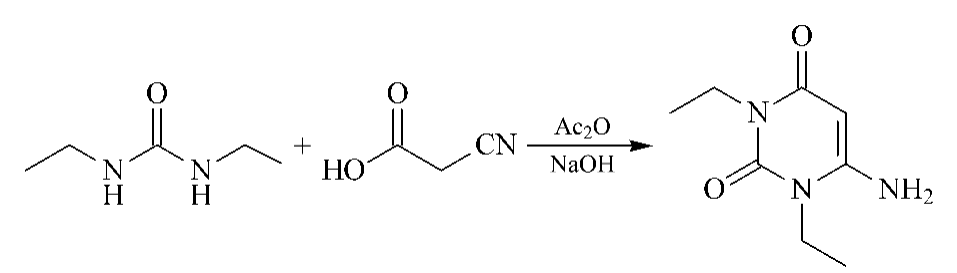
中间体A1合成路线

在我们前期的研究中,中间体A1的一个杂质已经被鉴定出并进行了表征,如图3所示,其结构被鉴定为(*E*)*-N*-乙基-2-氰基-3-乙氨基-2-丁烯酰胺,此为中间体A1的降解杂质^[20]^。在对合成过程中的关键参数进行研究和分析时,发现A1中还存在另外一个未知杂质。因此,我们继续展开研究以确定其结构,并对其进行鉴定及表征。

**图 3:**
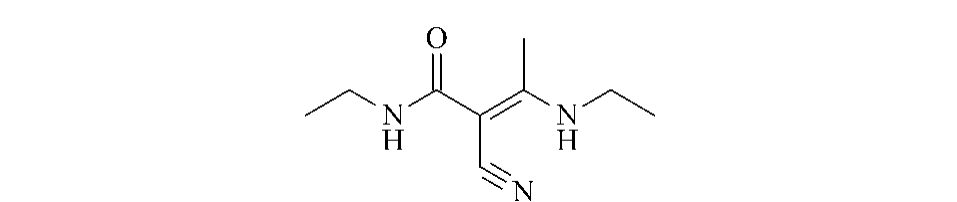
(*E*)*-N*-乙基-2-氰基-3-乙氨基-2-丁烯酰胺的结构

## 1 实验部分

### 1.1 仪器与试剂

高效液相色谱仪:配备SPD-10 Avp检测器的LC-20AD系统(日本Shimadzu);制备型高效液相色谱仪:KNAUER-AZURA HPLC系统(德国Knauer);高分辨率质谱(HRMS): 1200RRLC-6520 Accurate-Mass Q-TOF(美国安捷伦),其中ESI为正离子模式。核磁共振(NMR)分析仪:Bruker AⅧ 600 M(美国Bruker);单晶衍射仪:Bruker-apex2 X射线衍射仪(美国Bruker)。

伊曲茶碱中间体:山东新华制药股份有限公司(中国淄博)生产;色谱级乙腈、二甲基亚砜(DMSO)购自德国Merck;水通过Milli-Q水净化系统(美国Millipore)进行净化。

### 1.2 样品制备

称取0.4 g中间体A1放入50 mL的烧杯中,依次加入8 mL水、8 mL乙腈,超声溶解,然后通过0.45 μm有机膜过滤,得到测试样品溶液。

### 1.3 HPLC条件

分析型HPLC Agilent Zorbax C18色谱柱(150 mm×4.6 mm, 5 μm);柱温:35 ℃;流动相:乙腈(A)和水(B);流速:1.0 mL/min;梯度洗脱(*t*_min_/A:B)=*t*_0_/20:80, *t*_15_/60:40, *t*_20_~*t*_50_/90:10;检测波长:268 nm。

制备型HPLC Ceres B制备柱(250 mm×30 mm, 7 μm);流动相:乙腈-水(30/70, v/v);流速:30 mL/min;检测波长:268 nm。

### 1.4 核磁共振、质谱分析条件

NMR:将样品(氢谱:1 mg;碳谱:10 mg)放入清洁干燥的样品管中,加入1 mL DMSO溶解后将样品管密封好。将样品管插入转子,调整样品管的高度,样品管插入深度与量筒的底部相平。将转子按序列放入进样器,设置样品检测参数,开始测试。测试完成后,对图谱进行处理。

HRMS:将样品(10 mg)加入25 mL容量瓶中,用甲醇稀释至刻度,用0.22 μm的有机滤膜过滤,得到样品,备用。设置参数,进样,开始测试。测试完成后,对图谱进行处理,分析检测结果。

### 1.5 单晶X射线衍射条件

单晶制备:于50 mL单口瓶中加入1 g伊曲茶碱中间体A1、20 mL乙醇,搅拌加热至回流,待完全溶解,关掉热源,冷却至室温后,继续搅拌析晶2 h。抽滤干燥得伊曲茶碱中间体A1晶体。

检测条件:Mo-Kα辐射(*λ*=71.073 pm), 296 K。

## 2 结果与讨论

### 2.1 杂质分析

通过高效液相色谱对伊曲茶碱中间体A1进行有关物质分析,在保留时间6.82 min和20.22 min处有两个较大的杂质峰(见图4)。在前期的研究^[20]^中,保留时间20.22 min处的杂质已经被鉴定出并进行了表征, 保留时间6.82 min处杂质需要进一步分离、鉴定。

**图 4:**
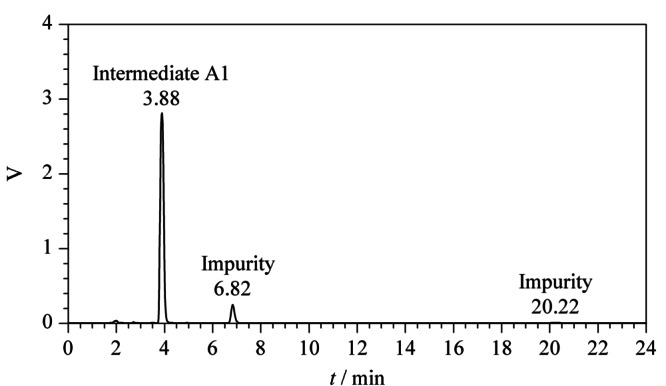
中间体A1相关物质的HPLC图

### 2.2 制备型HPLC分离、制备杂质

在1.3节制备型HPLC色谱条件下分离中间体A1及相关物质。从图5可以看出,中间体A1和杂质分离度较好,收集保留时间为5.80 min处的馏分(经薄层色谱及分析型HPLC分析确定为目标物),然后用二氯甲烷萃取,减压浓缩,干燥,得到淡黄色物料。

**图 5:**
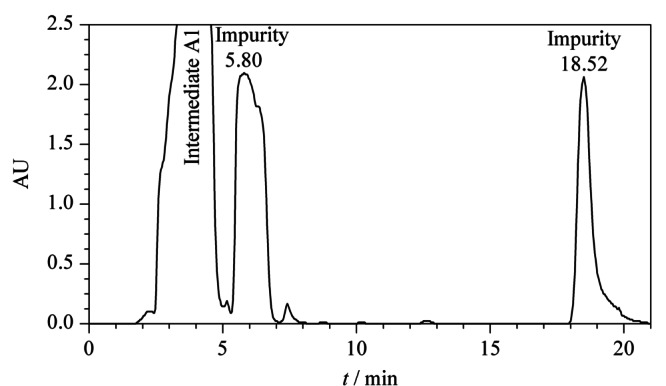
中间体A1相关物质的制备HPLC图

### 2.3 杂质的表征

从图6可以看出,杂质的HRMS图显示了质子化分子[M-H]^-^在*m/z* 253.19处,与中间体A1的质谱(*m/z* 183.1008)明显不同。

**图 6:**
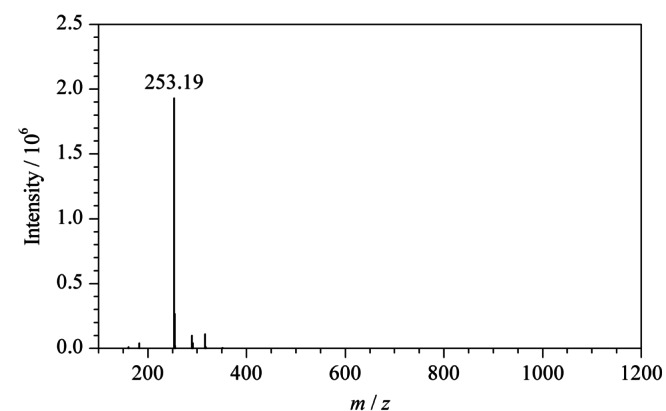
杂质的HRMS图

杂质的NMR光谱图见图7。

**图 7:**
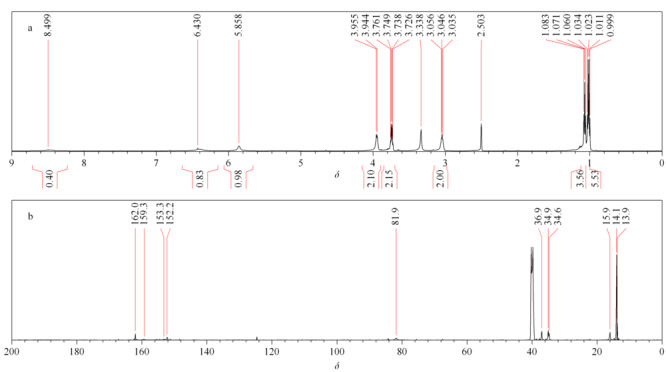
杂质的(a)^1^H NMR谱和(b)^13^C NMR谱

杂质^1^H-NMR:^1^H-NMR (600 MHz, DMSO): *δ* 1.01 (q, *J*=6.9 Hz, 3H), 1.02 (q, *J*=6.9 Hz, 3H), 1.07 (t, *J*=6.9 Hz, 3H), 3.04 (p, *J*=6.8 Hz, 2H), 3.74 (q, *J*=7.0 Hz, 2H), 3.94 (q, *J*=7.1 Hz, 2H), 5.85 (s, 1H)。

杂质^13^C-NMR (150 MHz, DMSO): *δ*13.9, 14.1, 15.9, 34.6, 34.9, 36.9, 81.9, 152.2, 153.3, 159.3, 162.0。

基于上述表征,该杂质比中间产物A1多一个甲基和一个亚甲基,杂质的分子骨架具有11个碳,这与具有8个碳的A1的分子骨架不一致。因此,可以得出结论,杂质是中间产物A1的副产物。

为了进一步确定杂质的结构,使用异核单量子相干谱(HSQC)和异核多碳相关谱(HMBC)(见图8)来表征氢和碳。根据HSQC, H(*δ*1.02)与H-5(*δ*3.74)相关,并且与C-6(*δ*13.9)相关,因此被确认为H-6。H(*δ*1.01)与H-10(*δ*3.04)相关,并且与C-11(*δ*15.9)相关,被确认为H-11。H(*δ*1.07)与H-7(*δ*3.94)相关,并且与C-8(*δ*14.1)相关,被确认为H-8。H(*δ*3.74)与H-6(*δ*1.02)相关,并且与C-5(*δ*34.9)相关,被确认为H-5。H(*δ*3.04)与H-11(*δ*1.01)相关,并且与C-10(*δ*34.6)相关,被确认为H-10。H(*δ*3.94)与H-8(*δ*1.07)相关,并且与C-7(*δ*36.9)相关,已确认为H-7。H(*δ*5.85)与C-3(*δ*81.9)相关,被确认为H-3。

**图 8:**
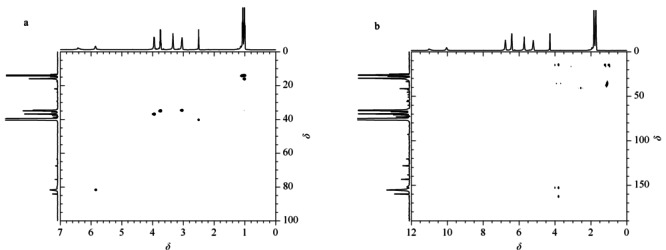
杂质的(a)HSQC和(b)HMBC NMR谱图

根据化学位移和相应的氢光谱,杂质的结构包含3个伯碳、3个仲碳、1个叔碳和4个季碳。在HMBC光谱中,伯碳峰(*δ*15.9)与H-11(*δ*1.01)相关,而与H-10(*δ*3.04)远程相关,因此是甲基C-11。C(*δ*14.1)与H-8(*δ*1.07)相关,而与H-7(d3.94)远程相关,为甲基C-8。C(*δ*13.9)与H-6(*δ*1.02)相关,而与H-5(*δ*3.74)远程相关,因此为甲基C-6。根据HMBC光谱,仲碳峰(*δ*36.9)与H-7(*δ*3.94)相关,而与H-8(*δ*1.07)远程相关,因此被确认为亚甲基C-7。C(*δ*34.9)与H-5(*δ*3.74)相关,而与H-6(*δ*1.02)远程相关,被确认为亚甲基C-5。C(*δ*34.6)与H-10(*δ*3.04)相关,而与H-11(*δ*1.01)远程相关,为亚甲基C-10。叔碳峰(*δ*81.9)与H-3(*δ*5.85)相关,与HMBC光谱无关,已确认为C-3。季碳峰(*δ*162.0)与HMBC谱图中H-7(*δ*3.94)远程相关,确认为C-4。HMBC谱显示C(*δ*159.3)与H-10(*δ*3.04)密切相关,确认为C-9。C(*δ*153.3)与H-7(*δ*3.94)和H-5(*δ*3.74)密切相关,确认为C-1。C(*δ*152.2)与H-5(*δ*3.74)远程相关,被确认为C-2。综上所述,推测该结构为1-(1,3-二乙基-2,6-二氧-1,2,3,6-四氢嘧啶-4-基)-3-乙基脲(见表1)。

**表 1:** 杂质的HSQC和HMBC核磁共振数据

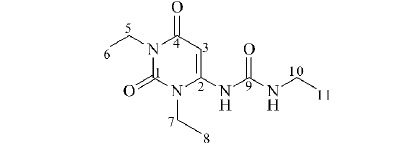				
Structure				
No. of C	*δ*_C_/*δ*_H_	HSQC	HMBC	C
1	153.3	-	H-7, 5	4°
2	152.2	-	H-5	4°
3	81.9/5.85	H-3	-	3°
4	162.0	-	H-7	4°
5	34.9/3.74	H-5	H-6	2°
6	13.9/1.02	H-6	H-5	1°
7	36.9/3.94	H-7	H-8	2°
8	14.1/1.07	H-8	H-7	1°
9	159.3	-	H-10	4°
10	34.6/3.04	H-10	H-11	2°
11	15.9/1.01	H-11	H-10	1°

为了进一步验证和确定物质的空间结构,用单晶X射线衍射对分离出的杂质进行了表征,表征结果见表2。通过数据分析及单晶衍射结构(见图9) 分析,进一步确定其结构为1-(1,3-二乙基-2,6-二氧-1,2,3,6-四氢嘧啶-4-基)-3-乙基脲。

**表 2:** 杂质的晶体数据

Parameter	Content
Empirical formula	C_11_H_18_N_4_O_3_
Formula weight	254.29
Temperature/K	296(2)
Crystal system	monoclinic
Space group	P2_1_/n
*a*/nm	0.5076(3)
*b*/nm	1.8133(10)
*c*/nm	1.3835(8)
*α*/°	90
*β*/°	95.670(9)
*γ*/°	90
Volume/nm^3^	1.267(12)
*Z*	4
Reflections collected	11865
Final *R* indices [*I*>=2σ (I)]	*R*_1_=0.0623, wR_2_=0.1617
Final *R* indices [all data]	*R*_1_=0.1589, wR_2_=0.2145

*a*, *b*, *c*: edge length of unit cell; *α*, *β*, *γ*: the angle between the edges of a unit cell; *Z*: number of molecules in unit cell; *R*_1_: non weighted consistency factor; wR_2_: weight consistency factor.

**图 9:**
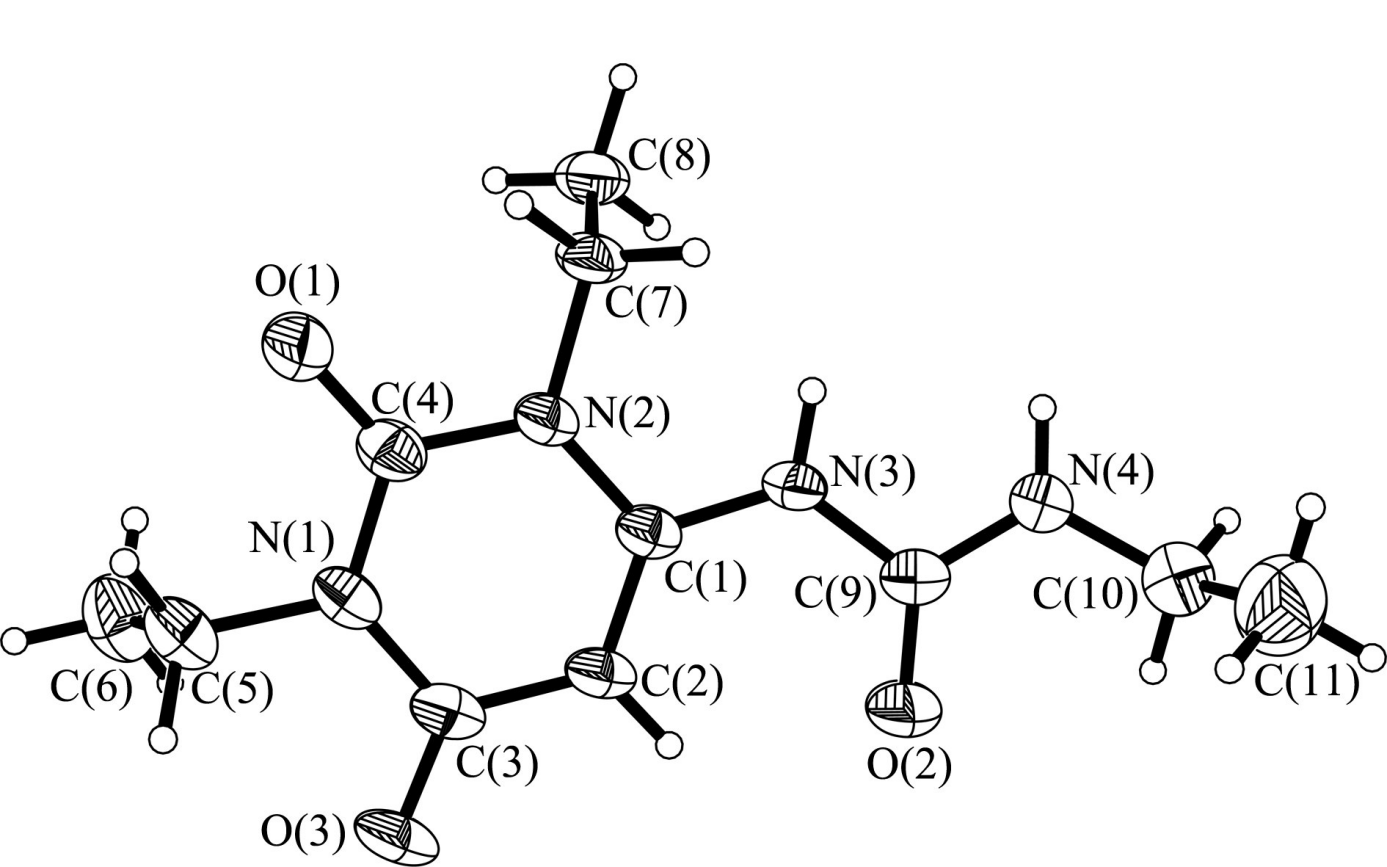
杂质的单晶X射线衍射结构

### 2.4 杂质的形成机理

在合成中间体A1时使用的原料是过量的,因此我们推断杂质的形成机理可能如下:氰基乙酸与二乙基脲、乙酸酐进行缩合反应得到中间体A1的过程(见图2)中,过量的二乙基脲继续与A1发生酰胺化反应生成1-(1,3-二乙基-2,6-二氧-1,2,3,6-四氢嘧啶-4-基)-3-乙基脲,即生成了此杂质(见图10)。此杂质为中间体A1副产物,与前期研究^[20]^中发现的中间体A1降解杂质(*E*)*-N*-乙基-2-氰基-3-乙氨基-2-丁烯酰胺形成机理虽然不同,但同样都是在高温条件下形成的。

**图 10:**
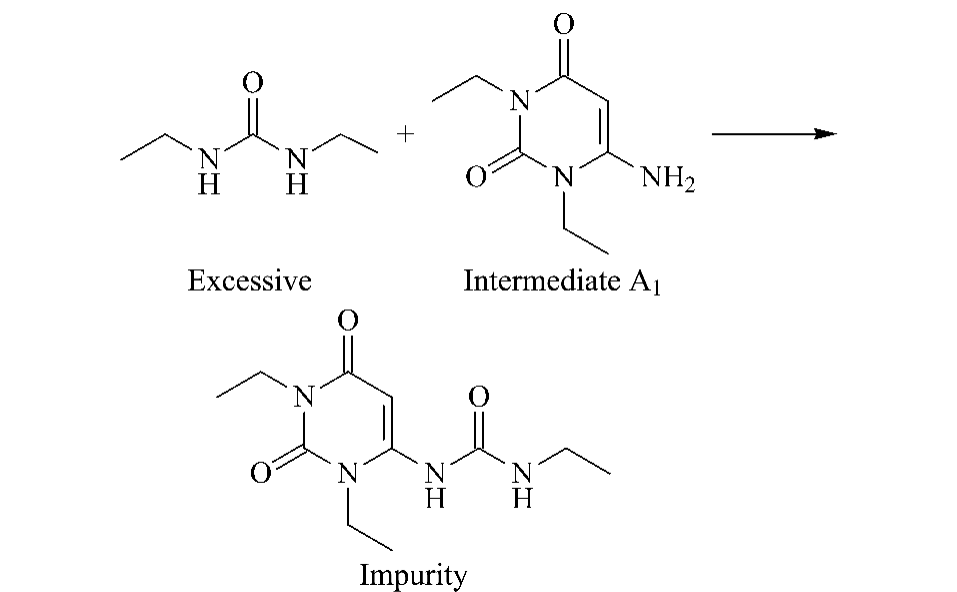
推测的杂质反应机理

## 3 结论

本研究采用制备型HPLC分离伊曲茶碱中间体A1中的杂质。通过HRMS、NMR、2D NMR和单晶X射线衍射分析,杂质结构确定为1-(1,3-二乙基-2,6-二氧-1,2,3,6-四氢嘧啶-4-基)-3-乙基脲。推测其反应机理为:由于在碱性条件下或高温下合成中间体A1时过量的二乙基脲而产生了中间体A1的副产物。该杂质的结构可能会对伊曲茶碱的安全性和有效性产生影响。为了确保伊曲茶碱的质量,在生产过程中需要对该杂质的含量进行控制。
